# Effect of Wine Matrix Composition on the Quantification of Volatile Sulfur Compounds by Headspace Solid-Phase Microextraction-Gas Chromatography-Pulsed Flame Photometric Detection

**DOI:** 10.3390/molecules24183320

**Published:** 2019-09-12

**Authors:** Peter M. Davis, Michael C. Qian

**Affiliations:** 1 Department of Food Science & Technology, Oregon State University, Corvallis, OR 97331, USA; yungdoufu@gmail.com; 2 Oregon Wine Research Institute, Oregon State University, Corvallis, OR 97331, USA

**Keywords:** wine matrix, volatile sulfur compounds, HS-SPME, GC-PFPD

## Abstract

The analysis of volatile sulfur compounds using headspace solid-phase microextraction (HS-SPME) is heavily influenced by matrix effects. The effects of a wine matrix, both non-volatile and volatile components (other than ethanol) were studied on the analysis of several common sulfur volatiles found in wine, including hydrogen sulfide (H_2_S), methanethiol (MeSH), dimethyl sulfide (DMS), dimethyl disulfide (DMDS), dimethyl trisulfide (DMTS), diethyl disulfide (DEDS), methyl thioacetate (MeSOAc), and ethyl thioacetate (EtSOAc). Varying levels of devolatilized wine and common wine volatiles (acids, esters, alcohols) were added to synthetic wine samples to act as matrices. Sulfur standards were added and analyzed using gas chromatography with pulsed-flame photometric detection (GC-PFPD). Five internal standards were used to find best representatives of each compound despite matrix effects. Sensitivity remained stable with the addition of devolatilized wine, while addition of volatile components decreased sensitivity. DMS was found to be best measured against EMS; DMDS and the thioacetates were best measured against DES; H_2_S, MeSH, DEDS, and DMTS were best measured against DIDS. The method was used to quantitate the volatile sulfur compounds in 21 wines with various ethanol contents and volatile profiles.

## 1. Introduction

Volatile sulfur compounds (VSC), including H_2_S, methanethiol, ethanethiol, thiol esters dimethyl sulfide, dimethyl disulfide, as well as dimethyl trisulfide are frequently present in wine. These VSCs pose problems for winemakers as they exhibit off-odors of onion, garlic, cabbage, cheese, and rotten egg even at very low concentrations in wine, due to their very low sensory thresholds [1]. Wine makers need to know their concentrations at various stages of wine making process so proper mitigation actions can be taken. However, the analysis of VSCs is challenging because of their high volatility and low concentrations [2,3].

VSCs analysis typically needs to isolate these compounds from the sample, then separate them by gas chromatography before detection and quantification. Many conventional extraction techniques such as solvent extraction, static headspace sampling, or purge-and-trap are not quite suitable for the analysis of VSCs in wine. Solvent extraction causes loss of analytes during the concentration stage, particularly compounds with high volatility; headspace sampling often does not provide insufficient sensitivity for trace components; and purge-trap has great potential of thermal artifact formation. In addition, alcohols in wine further complicate the extraction and concentration.

The solid-phase micro-extraction (SPME) technique was introduced to the scientific community in 1993 and has since been widely adopted for a wide range of volatile analysis. Compared to traditional methods such as purge-and-trap, liquid-liquid extraction, and other sample handling techniques, the SPME technique offers many advantages. Along with being simple to use and relatively inexpensive, the SPME technique requires little overall sample preparation time by not requiring solvent extraction and allows characterization of the headspace in contact with the sample. Since the compounds are extracted in a confined container, loss of highly volatile compounds such as hydrogen sulfide and methanethiol can be eliminated. Decomposition and artifact formation can be minimized.

SPME can effectively extract and concentrate aroma compounds, and provide high sensitivity with minimum artifact formation. With the use of SPME fibers, sample preparation can be completed in minimal time. In addition, SPME equipment can be automated. While there are a growing number of available fiber coatings, the Carboxen-polydimethylsiloxane (CAR-PDMS) fiber has repeatedly demonstrated its exceptional ability to extract sulfur compounds, including methanethiol and dimethyl sulfide, from food and wine samples. The process of concentration with the CAR-PDMS fiber is adsorption of small molecules into micro-pores by the Carboxen phase in addition to absorption by the PDMS coating, lending to its greater capacity for extracting highly volatile, low molecular weight VSCs.

VSCs can be analyzed by GC, and a sulfur-specific detector such as flame photometric detector (FPD), sulfur chemiluminescence detector (SCD), or a pulsed flame photometric detector (PFPD) can be used for sensitive detection. The PFPD was developed in the early 1990′s by Dr. Aviv Amirav from Tel Aviv University in Israel. Unlike the traditional flame photometric detector (FPD) which has a continuous flame, the PFPD is based on a pulsed flame for the generation of flame chemiluminescence. The detector operates with a fuel rich mixture of hydrogen and air, which is then ignited and propagated into a combustion chamber at a rate of three to four times per second where the flame front extinguishes. Specific elements have their own emission profile, carbon light emissions and the emissions from the hydrogen/oxygen combustion flame are complete in two to three milliseconds, while sulfur emissions begin at a relatively later time after combustion. Therefore, a timed “gate delay” can selectively allow for only emissions of sulfur to be integrated, producing a clean chromatogram. This timed “gate delay” greatly improves the sensitivity; the PFPD can detect sulfur-containing compounds at a much lower detection limit than many other methods of detection.

The combination of HS-SPME with GC-PFPD greatly enhances the ability to successfully extract and detect VSCs in wine at low concentrations. Nevertheless, the analysis of VSCs in wine is anything but straightforward, as the wine matrix composition, both nonvolatile and volatiles, including ethanol, can affect VSC extraction and analysis [4,5]. Different sulfur volatiles are not necessarily affected in the same way by the matrix [6].

The wine matrix is very complex, containing many different chemical classes and species including pigments, phenolics, acids, polysaccharides, proteins, alcohols, as well as volatile aromas. Pigments and phenolics act as antioxidants in wine [7,8], they can also prevent flavor from release [9] or react with analytes [10]. The flavor binding is also true for other components of wine matrix such as protein and polysaccharides. Nonvolatile matrix is typically separated from volatiles when flavor trapping [11,12] and solid-phase extraction [13,14] were used to concentrate the volatiles.

Although HS-SPME has been adopted as a quick and easy method for flavor analysis [15], it has been facing many criticisms [3,16]. It has been shown that matrix has a significant effect on the extraction of sulfur volatiles using HS-SPME. Some attribute a loss of sensitivity to competition for limited adsorption space on the SPME fiber [5,6], while others have seen the same effects using static headspace analysis [4,17]. In the latter case, ethanol was suggested to act as a co-solvent for the volatile compounds, limiting their ability to enter the headspace. Furthermore, not all sulfur volatiles are affected equally by matrix parameters [18].

This study aims to understand the influences of wine non-volatile and volatile components other than ethanol on the analysis of sulfur compounds using HS-SPME-GC-PFPD, and to develop a method to compensate for the matrix effect by selecting internal standards that behave most similarly to the target analytes. Six devolatilized wines (DVWs) served as non-volatile matrix standards, and mixtures of most-prominent non-sulfur-containing volatiles in wine were used as volatile matrix standards, including acids, alcohols, and esters, based on reported ranges in wines [19]. In addition, the method was used to analyze volatile sulfur compounds in 21 different wines with various volatile and nonvolatile composition.

## 2. Results and Discussion

### 2.1. Non-Volatile Matrix Effects

**Figure 1:**
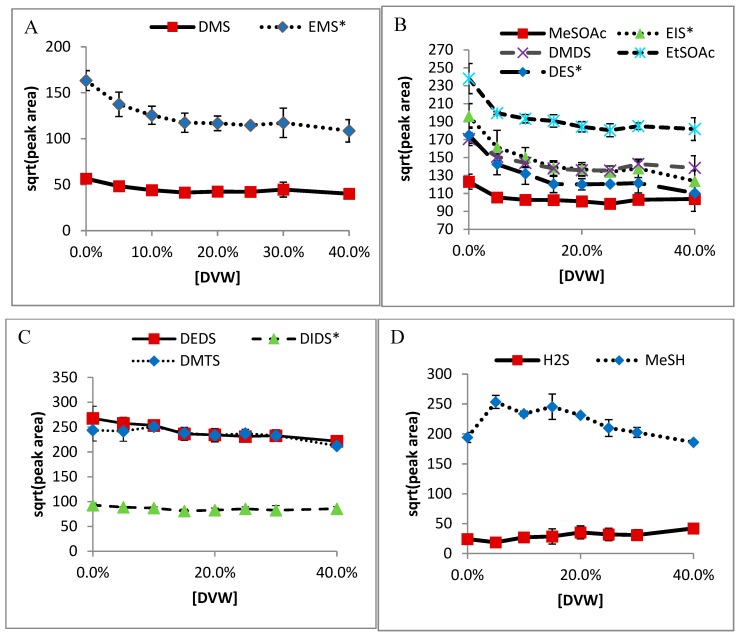
Effect of devolatilized Chardonnay wine matrix (DVW) on HS-SPME GC-PFPD analysis of: (**A**) DMS and EMS (IS), (**B**)MeSOAc, DMDS, EtSOAc, DES (IS), and EIS (IS), (**C**) DEDS, DMTS, and DIDS (IS), (**D**) H_2_S, and MeSH.

**Figure 2:**
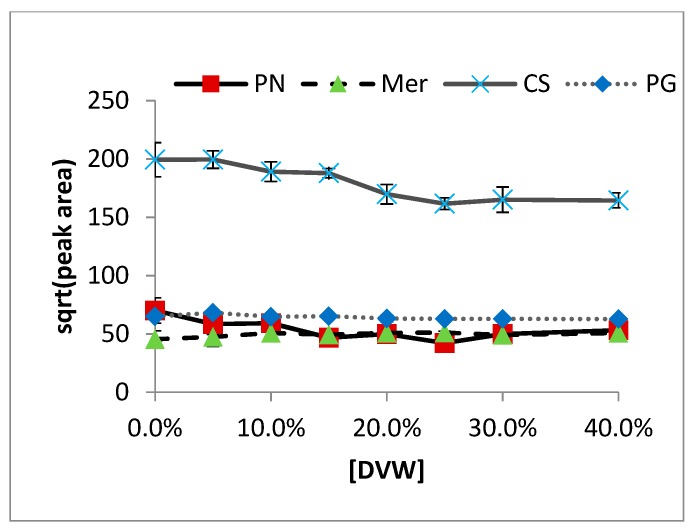
Effects of various DVW matrices on DMS extraction: PN = Pinot noir, Mer = Merlot, CS = Cabernet Sauvignon, PG = Pinot Grigio.

Results of DVW effects on sulfur extraction from chardonnay wine are shown in Figure 1. Though a very gradual decrease can be seen in all compounds, there is very little effect seen in chardonnay wine. Similar curves are seen for all other wine matrices (Figure 2). The slight decrease as DVW content rises is likely due to a decrease in salt in the system, as 40% DVW reduces salt water content to less than 6 mL.

**Figure 3:**
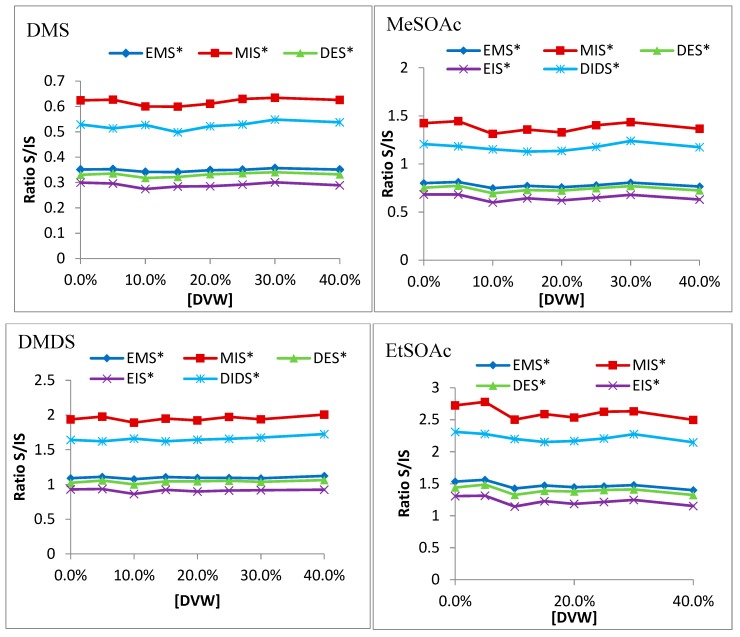

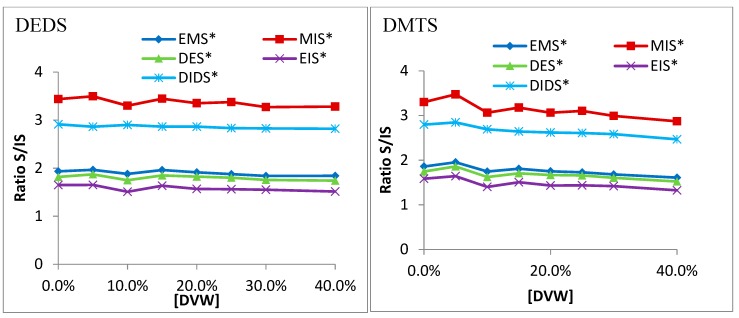
Analyte-to-internal-standard ratios to all five internal standards in merlot DVW of DMS, MeSOAc, DMDS, EtSOAc, DEDS, and DMTS.

More important are the ratios of analytes to internal standards (Figure 3), which gauge how closely the intended internal standard resembles the analyte in question. DMS, DMDS, MeSOAc, EtSOAc, DEDS, and DMTS all show very consistent ratios as DVW concentration increases. DMS closely matches EMS; DMDS, MeSOAc, and EtSOAc all closely follow EIS, DES, and EMS, though ethanol-effect studies have suggested EIS is ideal. DEDS and DMTS are well-represented by DIDS.

### 2.2. Volatile-Matrix Effects

**Figure 4:**
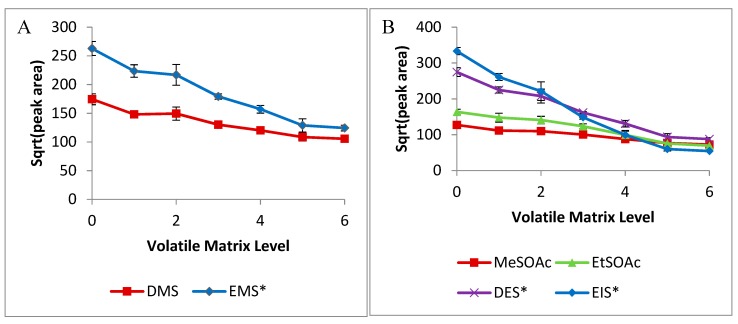

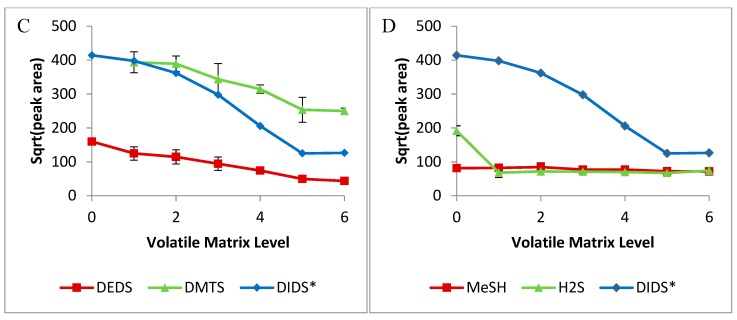
Effects of volatile acids, alcohols, and esters (across reported ranges in wine) on SPME adsorption of: (**A**) DMS and EMS (IS); (**B**) MeSOAc, EtSOAc, DES (IS), EIS (IS); (**C**) DEDS, DMTS, and DIDS (IS); (**D**) MeSH, H_2_S, and DIDS (IS).

The analyses of volatile sulfur compounds with varying levels of other (non-sulfur) volatiles are shown in Figure 4. Data is arranged by volatile-matrix level, ranging from 0 (no additional volatiles added) to 6. These correlate with the aforementioned concentrations of each compound in each set (acids, esters, alcohols). Analysis of the total mixture was performed foremost, in order to gauge effects; the total mixture most closely reflects that of a wine, which would not be completely deficient in one category. Thus, within a wine, the volatiles would have a cumulative effect as measured. Results from this total mixture best exemplify the effects of other volatile constituents on SPME adsorption of sulfur compounds.

As seen with ethanol, a strong decrease in the adsorption of volatile sulfur compounds is seen with increasing volatile-profile concentration. This suggests a competitive mechanism, as the volatile matrix components will fill the headspace and adhere to the fiber. The concentrations of each volatile added are insufficient to act as co-solvents as ethanol might, though may affect the equilibrium of volatiles in the headspace as more compounds become present [4,17].

**Figure 5:**
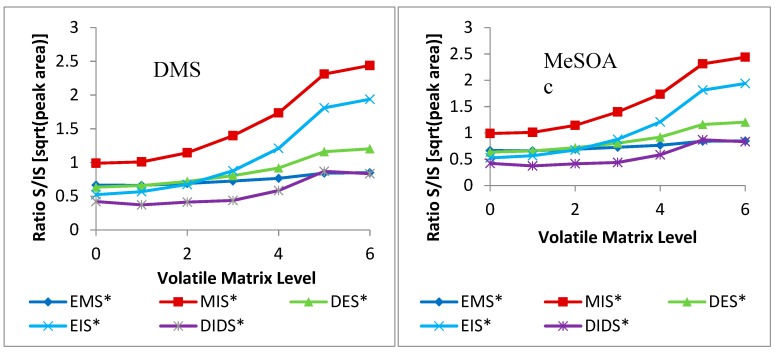

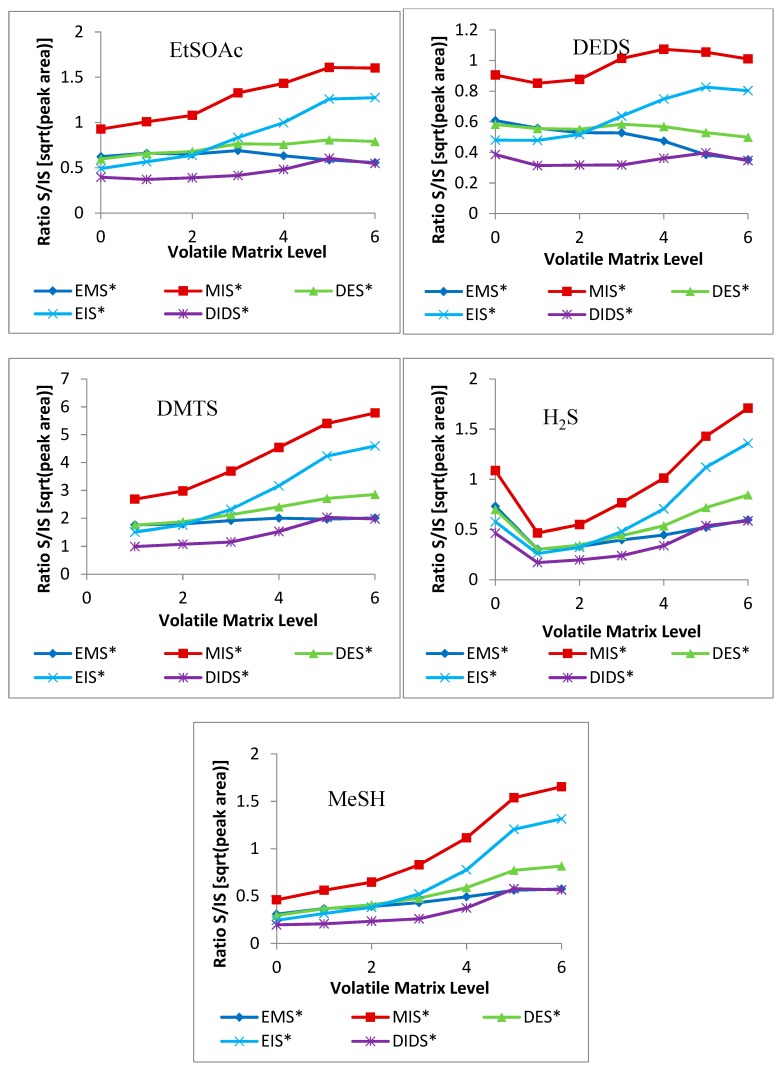
Effect of volatile matrix on analyte-to-internal-standard ratios against all five internal standards of DMS, MeSOAc, EtSOAc, DEDS, DMTS, H_2_S, and MeSH.

The analyte-to-internal-standard ratios (Figure 5) showed high variation. DMS still closely follows EMS. In ethanol studies, MeSOAc and EtSOAc both resemble EIS and DES, suggesting they might be accurate internal standards. However, the volatile-matrix data suggests that EIS loses its similarity at higher concentrations of volatiles. While EMS seems to match closely with both, ethanol studies showed it did not function well with varied ethanol content. Thus, DES is the internal standard of choice for the thioacetates. DEDS and DMTS, similar to the thioacetates, show good correlation with EMS. However, ethanol studies also suggested that DIDS was the only viable internal standard. H_2_S and MeSH are not well-represented by any of the internal standards, though they seem to correlate with EMS and DIDS. EMS was not found to correlate well with shifting alcohol contents, however, so DIDS remains the most viable internal standard for both.

The analysis of sulfur compounds using HS-SPME is heavily influenced by the presence of other volatiles. Little effect is seen from non-volatile matrix components. Based on the results of both alcohol effects and volatile effect, ideal internal standards to compensate for variation of these parameters in multiple wines are EMS (for DMS), DES (for DMDS, MeSOAc, and EtSOAc), and DIDS (for H_2_S, MeSH, DEDS, and DMTS).

**Figure 6:**
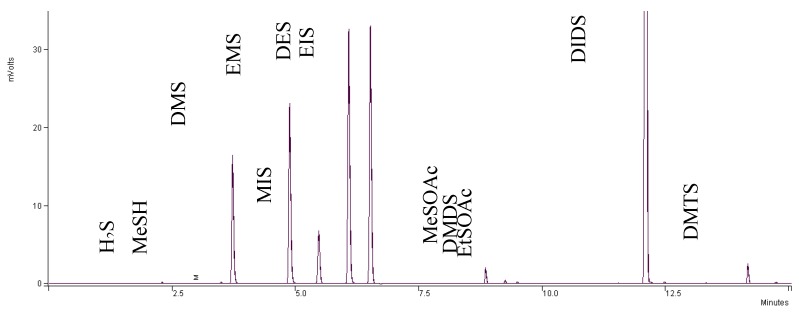
Representative GC-PFPD chromatogram of wine (cabernet sauvignon).

**Figure 7:**
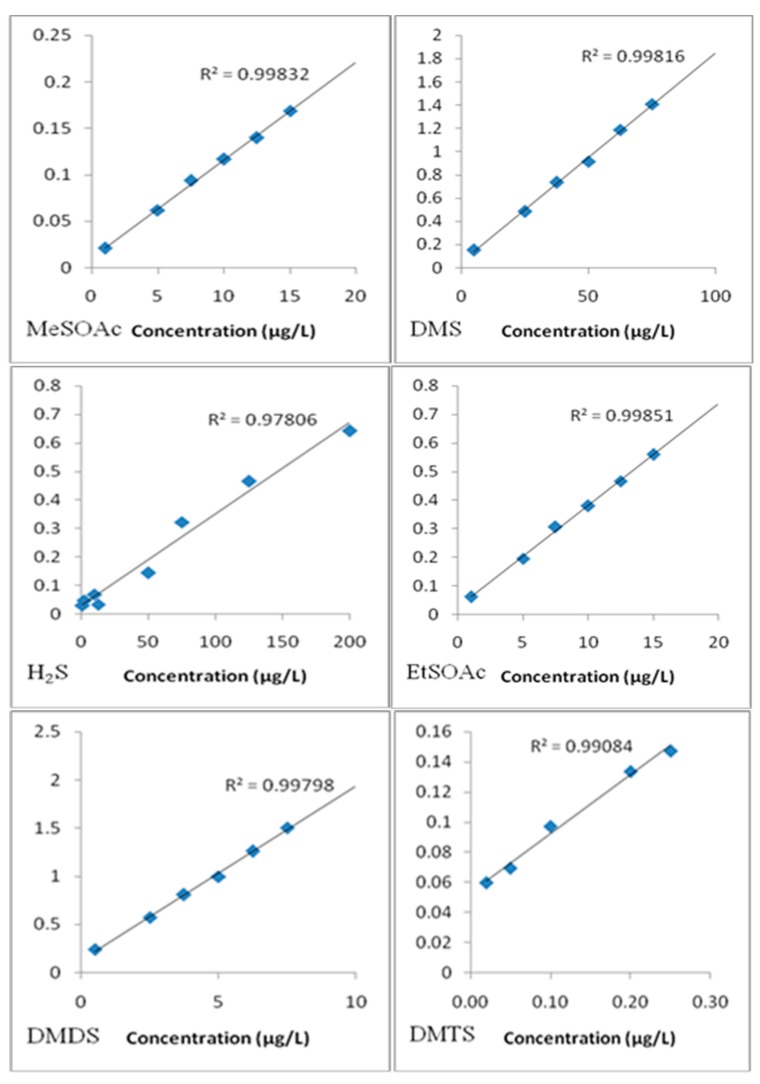
Calibration curves for some sulfur standards; y-axis = ratio of S:IS response (sqrt(peak area)); DMS (IS = EMS); DMDS, MeSOAc, EtSOAc (IS = DES); H_2_S, MeSH, DEDS, and DMTS (IS = DIDS).

A typical chromatogram for wine analysis is shown in Figure 6. This chromatogram represents a Cabernet Sauvignon wine. Standard curves were constructed to represent a range of concentrations near the odor threshold, as well as potential levels in wines (Table 1). Good linearity was seen for all curves, with R^2^ values greater than 0.99 for DMS, DMDS, MeSOAc, EtSOAc, DEDS, and DMTS. Highly volatile compounds H_2_S and MeSH achieved R^2^ values greater than 0.97 (Figure 7). 

The results of the analysis of 21 California wines are seen in Table 1. Traces of most compounds were found in all samples. Many wines had quantifiable levels of each sulfur compound. H_2_S was found frequently in trace amounts, though it may still be present in perceivable concentrations. Due to the broad range reported for its odor threshold value [20,21], it may be perceived at levels beneath its limit of detectability. White varietals like Chardonnay exhibit greater levels of H_2_S and MeSH than reds. DMS was found in slightly higher concentrations in red varietals, particularly Cabernet Sauvignon and Merlot. DMS and DMTS were the only compounds found consistently in all wines. Levels for DMS suggest a possible impact on the flavor of the wines, as concentrations slightly above the odor threshold are said to impart a beneficial fruity aroma [22].

## 3. Materials and Methods

### 3.1. Chemicals

Sodium sulfide, methanethiol (MeSH), dimethyl disulfide (DMDS), dimethyl trisulfide (DMTS), diisopropyl disulfide (DIDS), hexanoic acid, octanoic acid, phenethyl alcohol, 3-methyl-1-butanol, 2-methyl-1-propanol, ethyl acetate, 3-methyl-1-butyl acetate, ethyl hexanoate, and ethyl decanoate were from Sigma-Aldrich (St. Louis, MO, USA). Ethyl octanoate was from Eastman (Rochester, NY, USA). Methyl thioacetate (MeSOAc), ethyl thioacetate (EtSOAc), and diethyl sulfide (DES) were from Alfa-Aesar (Ward Hill, MA, USA). Ethyl methyl sulfide (EMS), dimethyl sulfide (DMS), diethyl disulfide (DEDS), methyl isopropyl sulfide (MIS), and ethyl isopropyl sulfide (EIS) were from TCI America (Portland, OR, USA). Methanol was from EMD Chemicals Inc. (Gibbstown, NJ, USA), l-tartaric acid from J.T. Baker (Phillipsburg, NJ, USA), and ethanol was from Koptec (King of Prussia, PA, USA).

### 3.2. Calibration of Sulfur Compounds

Hydrogen sulfide standards were prepared using equivalents of sodium sulfide (Na_2_S) dissolved in distilled water, and further diluted with cold (−15 °C) methanol. MeSH standards were prepared by bubbling the pure gas over cold methanol and recording gained mass. All other standards were prepared by dilution with cold methanol. A standard mixture (mix 1) was prepared containing DMS (3000 µg/L), MeSOAc (1285 µg/L), DMDS (218 µg/L), EtSOAc (564 µg/L), DEDS (55 µg/L), and DMTS (47 µg/L). Because MeSH readily oxidizes to DMDS, and the higher affinity for DMDS on the SPME fiber causes much greater peak responses, the two compounds were not calibrated simultaneously. A separate mixture (mix 2) was thus prepared containing MeSH (37 µg/L) and H_2_S (31 µg/L). A mixture containing EMS (5 mg/L), DES (1 mg/L), MIS (1.5 mg/L), and DIDS (25.9 µg/L) was used for internal standards. Calibration samples consisted of 2 mL synthetic wine (3.6 g/L tartaric acid) diluted to 10 mL with saturated salt water and ethanol, for a final ethanol content of 3%. Vials were flushed with argon and internal standards mixture (10 µL) and analyte calibration levels (20 µL) were added through the septum.

### 3.3. Volatile-Matrix Effect

Four separate sets of volatile-matrix standards were prepared; these consisted of acids (acetic, hexanoic, octanoic, decanoic), alcohols (2-methyl-1-propanol, 3-methyl-1-butanol, phenethyl alcohol), esters (ethyl acetate, 3-methyl-1-butyl acetate, ethyl hexanoate, ethyl octanoate, ethyl decanoate) and a total mixture of all three. Each set was prepared by diluting the respective compounds in cold (4 °C) ethanol. Final concentrations of each compound in the acid and alcohol mixtures (after added to synthetic wine to reflect base wine concentration) were 1, 2, 3, 4, 5, and 6 mg/L. Final concentrations of each compound in the ester mixture were 0.1, 0.25, 0.5, 1, 2, and 3 mg/L. Final concentrations of each compound in the total mixture of acids, alcohols, and esters, were the same as in their respective mixtures. The cumulative concentration of compounds in the highest level (level 6) of the total mixture consisted of four acids each at 6 mg/L, three alcohols each at 6 mg/L, and five esters each at 3 mg/L, thus 57 mg/L total.

### 3.4. Non-Volatile Matrix Effect

Three wines were supplied by E&J Gallo Winery (Modesto, CA, USA) to be devolatilized, consisting of: Louis Martini Cabernet Sauvignon (2009), Gallo Family Vineyards Pinot Grigio (blend), and Dancing Bull Sauvignon Blanc (2009). A pinot noir (2007) and chardonnay (2007) from Argyle Winery (Dundee, OR, USA) and a merlot (2004) from Hogue Cellars (Prosser, WA, USA) were also used. Wines were devolatilized as follows: 300 mL of wine was boiled using a rotary evaporator (Büchi, Switzerland) under vacuum at 40 °C and 85 rpm. Each wine was boiled until 40% remained (120 mL), then distilled water was added back to original concentration. This ensured all volatile compounds had been evaporated, including ethanol.

### 3.5. Volatile Analysis

Samples were prepared in 20 mL deactivated screw-cap glass vials with Teflon-faced silicone septa. Devolatilized wine samples were prepared using varying levels of DVW, consisting of 0, 10, 15, 20, 25, 30, and 40% wine matrix. Ethanol (0.3 mL) and saturated salt water were added to reach a final volume of 10 mL, and final ethanol concentration of 3%. Vials were flushed gently with argon under low flow rate (barely disturbing the surface of the sample liquid) to avoid turbulence. All samples received 20 µL of standard mixture (mix 1 or 2) and 10 µL of IS mix (30 µL of methanolic solutions added in total). In the case of mix 2, all standards were introduced via syringe through the sample-vial septum to avoid oxygen contact. All standards were stored in the freezer (−15 °C).

Volatile-matrix samples consisted of 2 mL synthetic wine (3.6 g/L tartaric acid) at 15% ethanol. Salt water was added to reach a final volume of 10 mL and final ethanol concentration of 3%. Volatile compound sets (i.e., acids, alcohols, esters, or total) were added at 20 µL at each level, to reach final concentrations listed. In an effort to consolidate sulfur analysis, a combination of mix 1 and mix 2 was prepared containing all sulfur standards. However, because of the oxidation of MeSH to DMDS, DMDS was not measured. To each sample, 20 µL of sulfur-standards mix and 10 µL of internal standards mix was added, reaching a final addition of 50 µL standards. All standards were added via syringe to avoid oxygen intake.

### 3.6. Wine Samples

Wines were provided by E&J Gallo Winery. A total of 21 California wines were analyzed, consisting of 13 red and 8 white, 9 different varietals, and 2 blends. Wine samples were prepared by diluting 2 mL of wine to 10 mL with saturated salt water and adding 10 µL internal standard mix and 5 µL of 20 mg/L acetaldehyde to counteract SO_2_ [23].

### 3.7. SPME Conditions

The SPME fiber used was an 85 µm Carboxen-PDMS (Supelco, Bellafonte, PA, USA). The samples were equilibrated at 30 °C for 5 min and the extraction took place for 20 min with agitation at 250 rpm. Injection temperature was 300 °C. Samples were analyzed in triplicate.

### 3.8. GC-PFPD

Samples were run on a Varian CP-3800 gas chromatograph equipped with a pulsed-flame photometric detector (PFPD; Varian, Walnut Creek, CA, USA) based on the method published by Fang and Qian [23]. A DB-FFAP column (30m x 0.32mm x 1 µm, Agilent, Palo Alto, CA, USA) was used for separation. A temperature program was used for the GC oven: 35 °C for 3 min, ramped to 150 °C at 10 °C/min, held 5 min, ramped to 220 °C at 20 °C/min, held 3 min. Nitrogen was used as carrier gas at 2 mL/min flow rate. Detector temperature was 300 °C with 14 mL/min hydrogen, 17 mL/min air 1, and 10 mL/min air 2. The PFPD was operating in sulfur mode, with 6 ms gate delay and 20 ms gate width. Data analysis relied on square roots of peak areas.

## Figures and Tables

**Table 1:** Quantification of sulfur volatiles in 21 different wines (µg/L).

Varietal	Year	H2S	MeSH	DMS	MeSOAc	DMDS	EtSOAc	DEDS	DMTS
Chardonnay	2009	2.35 ± 0.29	5.64 ± 0.57	25.12 ± 0.60	6.03 ± 0.09	0.16 ± 0.01	<0.1	<0.01	0.18 ± 0.02
Chardonnay	blend	19.35 ± 4.51	7.07 ± 0.36	53.02 ± 0.12	<1	0.06 ± 0.01	<0.1	<0.01	0.18 ± 0.01
Chardonnay	blend	22.25 ± 5.52	6.85 ± 0.50	30.77 ± 0.57	6.67 ± 1.55	0.64 ± 0.08	<0.1	0.01 ± 0.001	0.32 ± 0.04
Moscato	2010	1.15 ± 0.23	<0.1	4.08 ± 0.04	24.02 ± 14.11	0.02 ± 0.01	2.88 ± 1.96	0.02 ± 0.004	0.03 ± 0.002
Pinot Gris	2010	8.82 ± 0.11	0.66 ± 0.08	25.44 ± 0.02	<1	<0.01	<0.1	<0.01	0.05 ± 0.004
Riesling	2009	1.11 ± 0.14	2.56 ± 0.05	11.48 ± 0.62	<1	0.01 ± 0.001	<0.1	<0.01	0.14 ± 0.03
Riesling	2009	5.47 ± 1.13	<0.1	14.53 ± 0.55	<1	<0.01	<0.1	<0.01	0.03 ± 0.002
Sauv. Blanc	2009	<1	<0.1	13.57 ± 1.33	15.19 ± 13.45	1.06 ± 0.02	5.00 ± 0.43	0.08 ± 0.003	0.04 ± 0.000
White (blend)	blend	25.39 ± 3.82	3.31 ± 0.41	17.84 ± 0.99	<1	<0.01	<0.1	<0.01	0.04 ± 0.004
Cab. Sauv.	2009	<1	1.25 ± 0.09	59.46 ± 0.01	21.38 ± 0.15	0.16 ± 0.03	0.71 ± 0.02	<0.01	0.18 ± 0.007
Cab. Sauv.	2008	<1	3.21 ± 0.28	84.51 ± 5.82	7.75 ± 0.17	0.27 ± 0.02	<0.1	<0.01	0.02 ± 0.002
Cab. Sauv.	2007	1.13 ± 0.20	1.95 ± 0.14	55.12 ± 0.01	2.07 ± 0.04	0.03 ± 0.001	<0.1	<0.01	0.02 ± 0.002
Cab. Sauv.	blend	<1	0.37 ± 0.03	42.04 ± 0.59	19.46 ± 0.04	<0.01	1.62 ± 0.13	<0.01	0.06 ± 0.004
Malbec	2009	<1	3.70 ± 0.44	45.22 ± 0.92	18.10 ± 0.16	0.48 ± 0.04	0.72 ± 0.01	<0.01	0.29 ± 0.01
Merlot	blend	<1	1.15 ± 0.03	42.24 ± 1.56	34.00 ± 0.23	<0.01	2.98 ± 0.02	<0.01	0.08 ± 0.01
Merlot	2009	<1	<0.1	34.99 ± 0.26	17.90 ± 0.31	<0.01	0.93 ± 0.08	<0.01	0.08 ± 0.002
Merlot	2008	<1	1.01 ± 0.05	51.26 ± 4.15	11.13 ± 4.12	0.54 ± 0.14	0.22 ± 0.31	<0.01	0.09 ± 0.003
Pinot noir	2009	<1	2.87 ± 0.51	19.44 ± 0.56	18.74 ± 0.46	0.08 ± 0.019	0.39 ± 0.01	<0.01	0.15 ± 0.04
Pinot Noir	blend	9.60 ± 0.76	1.19 ± 0.26	20.28 ± 0.25	22.56 ± 0.14	<0.01	1.52 ± 0.07	<0.01	0.10 ± 0.05
Zinfandel	2009	<1	2.13 ± 0.21	64.04 ± 0.11	29.28 ± 0.05	0.04 ± 0.000	2.43 ± 0.10	<0.01	0.23 ± 0.01
Red (blend)	blend	<1	1.52 ± 0.18	33.49 ± 1.89	42.95 ± 0.30	<0.01	5.68 ± 0.05	<0.01	0.13 ± 0.02
